# An Approach Based on HPLC-Fingerprint and Chemometrics to Quality Consistency Evaluation of *Matricaria chamomilla* L. Commercial Samples

**DOI:** 10.3389/fpls.2016.01561

**Published:** 2016-10-21

**Authors:** Agnieszka Viapiana, Wiktoria Struck-Lewicka, Pawel Konieczynski, Marek Wesolowski, Roman Kaliszan

**Affiliations:** ^1^Department of Analytical Chemistry, Medical University of GdanskGdansk, Poland; ^2^Department of Pharmaceutics and Pharmacodynamics, Medical University of GdanskGdansk, Poland

**Keywords:** plant polyphenols, *Matricaria chamomilla* L., chromatographic fingerprint, antioxidant activity, chemometric analysis

## Abstract

Chamomile has been used as an herbal medication since ancient times and is still popular because it contains various bioactive phytochemicals that could provide therapeutic effects. In this study, a simple and reliable HPLC method was developed to evaluate the quality consistency of nineteen chamomile samples through establishing a chromatographic fingerprint, quantification of phenolic compounds and determination of antioxidant activity. For fingerprint analysis, 12 peaks were selected as the common peaks to evaluate the similarities of commercial samples of chamomile obtained from different manufacturers. A similarity analysis was performed to assess the similarity/dissimilarity of chamomile samples where values varied from 0.868 to 0.990 what indicating that samples from different manufacturers were consistent. Additionally, simultaneous quantification of five phenolic acids (gallic, caffeic, syringic, p-coumaric, ferulic) and four flavonoids (rutin, myricetin, quercetin and keampferol) was performed to interpret the quality consistency. In quantitative analysis, the nine individual phenolic compounds showed good regression (*r* > 0.9975). Inter- and intra-day precisions for all analyzed compounds expressed as relative standard deviation (CV) ranged from 0.05% to 3.12%. Since flavonoids and other polyphenols are commonly recognized as natural antioxidants, the antioxidant activity of chamomile samples was evaluated using 1,1-diphenyl-2-picrylhydrazyl (DPPH) radical scavenging activity and ferric reducing/antioxidant power (FRAP) assay. Correlation analysis was used to assess the relationship between antioxidant activity and phenolic composition, and multivariate analysis (PCA and HCA) were applied to distinguish chamomile samples. Results shown in the study indicate high similarity of chamomile samples among them, widely spread in the market and commonly used by people as infusions or teas, as well as that there were no statistically significant differences among them, which in turn is a proof of high quality of commercially available samples of chamomile. The study indicated that the combination of chromatographic fingerprint and quantitative analysis can be readily utilized as a quality consistency method for chamomile and related medicinal preparations. Moreover, the applied strategy seems to be the most promising for the assessment of the investigated plant material.

## Introduction

Chamomile—*Matricaria chamomilla* L. has been used by humans for centuries (Gupta et al., 2010). It occurs naturally practically all over the world including Europe, Asia and Northern Africa, and it is also cultivated in Northern America. Applications include anti-inflammatory, immunomodulary, antidiabetic, acaricidal, antihyperglycemic, antimicrobial and antidepressant activity, amongst many others (McKay and Blumberg, 2006; Gupta et al., 2010; Strivastava et al., 2010; Amsterdam et al., 2012; Weidner et al., 2013). It must be stressed that traditional use of *Matricaria chamomilla* as Chamomillae romana flos has recently been included officially in the monographs of herbal medicinal products in the European Union (Chinou et al., 2014).

The biological activity of chamomile is associated with its essential oil and phenolic fractions (McKay and Blumberg, 2006). The major phenolic fraction of chamomile contains flavonoids, their aglycones and/or glycosides (Gupta et al., 2010; Nováková et al., 2010), coumarins (Kováčik and Repčák, 2008; Petrulová-Poracká et al., 2013) and phenolic acids (Nováková et al., 2010; Guimarães et al., 2013; Roby et al., 2013). Emphasis has been placed to these compounds due to their spasmolytic, antiphogistic and antioxidant activity. Some studies dealing with chamomile flowers are available in the literature, including reports on their phenolic composition (Mulinacci et al., 2000; Nováková et al., 2010; Guimarães et al., 2013) and antioxidant properties (Miliauskas et al., 2004; Katalinic et al., 2006; Barros et al., 2010).

Infusions and decoctions are the most commonly consumed preparations derived from chamomile. This wide use of herbal infusions makes it necessary to develop an effective method for the evaluation of quality consistency of herbal medicines and their preparations. At present, the fingerprint technique, especially chromatographic fingerprint, has become a powerful tool for quality control and has been internationally accepted for the evaluation and quality control of herbal medicines and preparations (Drug Administration, 2000; FDA, 2000; WHO, 2002).

The objective of this study was to develop a simple and efficient HPLC fingerprint method for quality consistency evaluation of chamomile samples obtained from different manufacturers in Poland. In this paper quality consistency of herbal medicines reflects variation in their chemical composition from batch to batch. The concentration of chemical constituents of herbal medicines can vary depending on several factors, such as botanical species, kinds of chemotypes, morphological parts of the plant, geographical area of cultivation, time of harvest, storage conditions, and others. Thus, the individual batches may differ significantly, for instance, in their medical activity and chemical stability. According to world regulations, it was assumed that such a consistency quality should comprise two elements. First of all, fingerprint analysis must be done, since it indicates authenticity and consistency of plant material, next similarity analysis, which shows mutual similarity among the samples. Hence, while performing fingerprint analysis it is possible to compare the chromatograms of samples and visually confirm similarity of the studied plant materials. However, it should be taken in mind that this type of analysis doesn't provide the knowledge about quantitative composition of samples. That's why there is a need to analyze quantitatively one or more active constituents of a given plant material. Because chamomile contains phenolic compounds that are commonly recognized as natural antioxidants, the most abundant phenolic acids (gallic, caffeic, syringic, p-coumaric, ferulic) and flavonoids (rutin, myricetin, quercetin, and keampferol) were determined along with the antioxidant activity of chamomile samples using DPPH and FRAP assays. The data set obtained after HPLC/UV determination underwent chemometric calculations including correction of retention time shifts (peaks alignment). In order to assess the similarity/dissimilarity of the chamomile samples, similarity analysis, principal component analysis (PCA) and hierarchical cluster analysis (HCA) were performed.

## Material and methods

### Plant material and reagents

Nineteen samples of chamomile were obtained from different manufacturers in Poland and are listed in Table 1 with their characterizations. The samples were homogenized at 20°C in a water-cooled grinder Knifetec 1095 (Foss Tecator, Höganäs, Sweden) and stored in desiccator which was protected from light.

**Table 1:** **The characterization of the *Matricaria chamomilla* L. samples and their similarities values**.

**No**	**Sample color and consistency**	**Manufacturer**	**Series**	**Similarity**
1	Yellow, granulated	Dary Natury/Koryciny	2014-01-01	0.910
2	Yellow, granulated	Dary Natury/Koryciny	2015-03-01	0.968
3	Light brown, fragmented	Herbarium/Ustroń	L22111331C	0.868
4	Light brown, fragmented	Herbarium/Ustroń	L14121331B	0.959
5	Light brown, fragmented	Herbarium/Ustroń	L14031431C	0.939
6	Brown, fragmented	Posti/Gniezno	L0160	0.933
7	Light brown, fragmented	Edal/Lisków	1901	0.971
8	Brown, fragmented	Herbapol/Kraków	01.10.12	0.899
9	Brown, fragmented	Herbapol/Lublin	02092012	0.879
10	Dark yellow, fragmented	Herbapol/Lublin	01032014	0.931
11	Light brown, fragmented	Herbapol/Lublin	01012014	0.933
12	Brown, fragmented	Vitax L/Warszawa	13297	0.948
13	Brown, fragmented	Phyto Pharm/Kleka	320005	0.955
14	Brown, fragmented	Flos/Mokrsko	1013	0.990
15	Brown, fragmented	Kawon/Gostyń	0602014	0.921
16	Yellow, granulated	Kawon/Gostyń	025.2013	0.929
17	Yellow, granulated	Kawon/Gostyń	003.2014	0.939
18	Light brown, fragmented	ApteoNatura/Warszawa	L:13/346P	0.970
19	Brown, fragmented	Biofix/Górki Małe	31.01.2016	0.970

Standards of phenolic acids—gallic, caffeic, syringic, p-coumaric, ferulic, and of flavonoids—rutin, myricetin, quercetin and kaempferol were purchased from ChromaDex (California, USA). HPLC grade methanol and acetonitrile were purchased from POCh (Gliwice, Poland) and J.T. Baker (Phillipsbusg, USA), respectively. Analytical grade ethanol, methanol, acetic acid, ferric chloride hexahydrate, 6-hydroxy-2,5,7,8-tetramethychroman-2-carboxylic acid (Trolox), 2,4,6-tris (2-pyridyl)-1,3,5-triazine (TPTZ), and 1,1-diphenyl-2-picrylhydrazol radical (DPPH) were purchased from POCh (Gliwice, Poland). Trifluoroacetic acid (TFA) was obtained from Sigma Aldrich (St. Louis, MO, USA). Redistillated water was prepared by triple distillation of water in a Destamat Bi-18 system (Heraeus Quarzglas, Hanau, Germany).

### Sample preparation

A 0.2 g sample of chamomile was accurately weighed and sonicated in a Polsonic ultrasonic bath (Warsaw, Poland) with 4 mL of methanol:water mixture (80:20, v/v) at 35°C for 30 min. This procedure was repeated three times and all recovered fractions were collected and diluted to 20 mL with methanol:water mixture (80:20, v/v). Prior to analysis, the extraction solution was filtered thought a 0.20 μm nylon membrane filter by Witko (Łódź, Poland) into a HPLC vial.

### Chromatographic analysis

Chromatographic separation and determination of phenolic compounds in chamomile extracts were performed using HPLC LaChrom system (Merck, Darmstadt, Germany) with a Hypersil Gold C18 column (250 × 4.6 mm; 5 μm) at 35°C—a system consisting of a L-7100 pump, L-7360 column compartment and L-7420 UV/Vis detector. Solvent A (0.05% TFA in acetonitrile) and solvent B (0.05% TFA in water) were used as a mobile phase. Optimized gradient elution was performed using the following program: 5–25% A (0–30 min), 25–40% A (30–40 min), 40–63% A (40–50 min) and 5% A (50–60 min). Before starting gradients runs, initial conditions were maintained over 20 min for column equilibration. The flow rate was set at 1.0 mL/min, injection volume at 20 μL, and UV detector wavelength at 254 nm. Identification of the samples' phenolic compounds was based on comparison of retention times with those of commercial standards.

### Linearity, LOD, LOQ, precision and accuracy

The linearity of the method was examined with standard solutions. A mixed stock solution (1 mg/mL) of nine phenolic compounds was prepared, along with fresh calibration working standard solutions in mixture of methanol:water (80:20, v/v) by appropriate dilution of the stock solution to yield six concentrations of phenolic compounds. Linearity was established by plotting the peak area (Y) vs. concentration (X) of each compound. The limits of detection (LOD) and quantification (LOQ) under the chromatographic conditions were calculated in μg/ml according to the following equations: LOD = 3.3S_xy_/b and LOQ = 10S_xy_/b, where S_xy_ is the standard deviation of the response, and b is the slope of the calibration curve. Intra- and inter-day variations (expressed as the coefficient of variation, CV), were chosen to determine the instrument precision of the assay developed. Intra-day precision was validated with a standard solution of assayed phenolic compounds three times within 1 day, while inter-day precision was validated with the same standard solution over three consecutive days. The peak areas and retention times of nine phenolic compounds were analyzed every 8 h within 48 h for the stability test.

### Antioxidant activity assays

The radical scavenging activity of chamomile extracts using DPPH assay was determined with the method developed by Tuberoso et al. (2010). Absorbance was measured at 517 nm using a Metertekh UV/Vis spectrophotometer (Nankang, Taiwan), and compared with a Trolox calibration curve. The results were expressed as mmol of Trolox per liter of extract (mmol TE/L).

Ferric reducing/antioxidant power (FRAP) assay was carried out using the method proposed by Benzie and Strain (1996), a technique based on the reduction at low pH of ferric 2,4,6-tris(pyridin-2-yl)-1,3,5-triazine (Fe^III^-TPTZ) to ferrous complex. The absorbance was measured at 593 nm and compared with the ferrous sulfate calibration curve. The results were expressed as mmol of Fe^2+^ per liter of extract (mmol Fe^2+^/L).

### Statistical analysis

All the assays were conducted in triplicate and results expressed as mean values ± standard deviation (SD). Analysis of variance (ANOVA) test, followed by Tukey test, was performed to check significant differences between samples due to different color, consistency, manufacturer or phenolic composition. Differences were considered significant at *p* < 0.05. A Pearson correlation was used to assess the relationship between antioxidant activity and phenolic composition and to establish the relative importance of phenolics for antioxidant activity. Calculations were performed using Statistica 7 software (StatSoft Inc., USA) on the basis of parametric tests with the level of significance of *p* < 0.05. With regard to data pretreatment procedure for the entire chromatograms obtained (fingerprints), peak alignment was performed using the Supervised Alignment method (SA). Next, similarity analysis was conducted for the aligned and unaligned chromatograms using a simulated mean reference chromatogram, which resulted in a list of correlation coefficient values calculated for each sample. Additionally, the variation of characteristic parameters among the chamomile samples was evaluated using principal component analysis (PCA) and hierarchical cluster analysis (HCA), which have been used as an initial step in many fingerprint studies (Abollino et al., 2011). Before unsupervised methods were applied, the data sets had undergone another data pretreatment procedure including column centering and autoscalation. All four data sets were centered, whereas autoscalation was applied for quantified data sets and data containing “common peaks.” Autoscalation was performed in order to treat all peaks equally important regardless their intensity (low or high). The results obtained from the aforementioned unsupervised multivariate methods were compared with similarity analysis results. Data calculations covering data pretreatment, unsupervised and supervised multivariate statistical methods were carried out using MATLAB 9.1 (The MathWorks, Inc., USA).

## Results and discussion

### Optimization of extraction conditions

An efficient extraction method is required for maximum extraction efficiency and well-separated chemical profiles with the lowest background signal from the matrices. The parameters affecting extraction efficiency, such as organic solvents (methanol and ethanol) in different concentrations (i.e., 60, 80, and 100%), extraction temperature (25, 30, and 35°C) and extraction time (10, 20, and 30 min) were all optimized. The best results were obtained with methanol:water mixture (80:20, v/v) at 35°C within 30 min. The optimum pre-treatment conditions presented in detail in Section Sample Preparation meant that the fingerprint analysis of chamomile was rapid, easy to perform, while still demonstrating high extraction efficiency of chamomile compositions.

### Optimization of HPLC conditions

In order to develop chromatographic fingerprints, different factors can be varied at different levels to increase the number of compounds separated (Alearts et al., 2014). In this study, to achieve the best separation and the most chemical information, the mobile phases (methanol, acetonitrile) with different modifiers (acetic and trifluoroacetic acids), column temperature (25, 30, and 35°C) and detection wavelength (254, 280, 320, and 370 nm) were optimized. After comparing both the number of separated peaks and their areas, 254 nm was selected as the detection wavelength. The effect of mobile phase composition with some modifiers on chromatographic separation was investigated under different gradient elution modes and acetonitrile was found to be the most appropriate. Finally, TFA (0.05%) was added to demonstrate a good resolution and satisfactory peak shape. Satisfactory separation was achieved in 60 min by gradient elution using the HPLC conditions described in Section Chromatographic Analysis.

### Method validation of quantitative analysis

A good correlation was found between peak area (Y) and concentration (X) (*r* > 0.997) for all phenolic compounds. The values of LODs and LOQs were less than 3.24 μg/mL and 9.89 μg/mL, respectively. Precision was acceptable, CV values ranging between 0.12% and 2.03% and between 0.11% and 3.03% for intra- and inter-day variations, respectively. For the stability test, retention CV was lower than 1.8% for peak area and 0.5% for retention time. Besides, peak areas and retention times of phenolics were found to be quite stable over 48 h. All results of method validation are summarized in Table 2.

**Table 2:** **Validation report of the methods for quantitation of phenolic compounds in chamomile samples (*n* = 6)**.

**Phenolic compounds**	**Gallic acid**	**Caffeic acid**	**Syringic acid**	**p-Coumaric acid**	**Ferulic acid**	**Rutin**	**Myricetin**	**Quercetin**	**Keampferol**
Range (μg/mL)	10–120	10–120	10–120	10–120	10–120	10–120	100–400	100–400	100–400
r	0.9999	0.9998	0.9997	0.9998	0.9998	0.9997	0.9975	0.9976	0.9980
LOD (μg/mL)	1.77	1.09	2.03	1.75	3.26	2.57	21.71	19.49	18.00
LOQ (μg/mL)	5.36	3.29	6.15	5.31	8.89	7.79	60.96	59.07	54.55
**INTRA-DAY**									
Nominal concentration (μg/mL)	70	70	70	70	70	70	250	250	250
Assayed concentration (μg/mL)	64.45	65.12	67.43	68.32	67.98	69.12	241.34	235	245
Recovery (%)	92.07	93.03	96.33	97.60	97.12	98.75	96.53	94.00	98.00
CV (%)	0.97	1.43	1.76	0.54	0.38	2.03	0.12	0.06	0.23
**INTER-DAY**									
Nominal concentration (μg/mL)	70	70	70	70	70	70	250	250	250
Assayed concentration (μg/mL)	63.02	64.35	66.54	68.98	67.12	68.95	218.00	242.00	241.00
Recovery (%)	90.03	91.93	95.06	98.55	95.89	98.50	87.20	96.68	96.40
CV (%)	1.54	2.14	3.03	1.65	0.87	3.12	0.11	0.05	0.33

### HPLC fingerprint of chamomile

**Figure 1:**
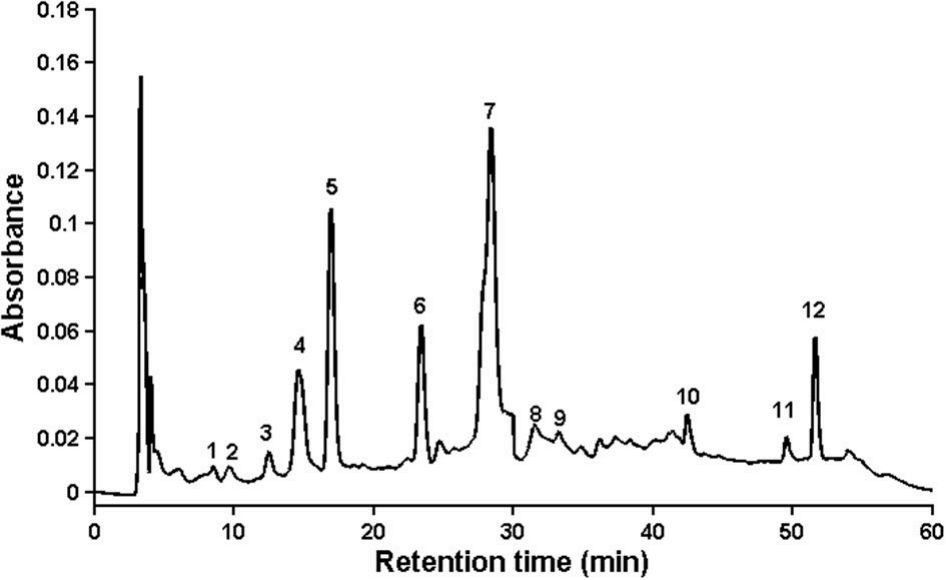
**Chromatographic fingerprint by HPLC/UV at 254 nm**.

In the study two aspects were applied, which were accepted by world regulations in order to investigate consistency quality of medicinal plant products (Drug Administration, 2000; FDA, 2000; WHO, 2002). First of them is based on fingerprint analysis, where “common peaks” should be found, which are present in each chromatogram, and these peaks should be well separated and have large areas. The second aspect relies on quantitative analysis, where retention times of standard substances are taken into account, so some bioactive compounds should be identified and assayed using calibration curves. Of course, it is desirable that quantified substances should belong to so called “common peaks,” but it is not always achievable, because at times they are difficult to be identified. For this reason, 19 samples of chamomile were analyzed, and approximately 60 peaks found in each individual sample. Peaks that existed in all samples with good resolution and reasonable heights were assigned as “common peaks” to express the characteristics of chamomile extracts. There were 12 common peaks in the fingerprint chromatogram, and the other peaks in early elution time (<5 min) were omitted because of their tendency to exhibit errors from peak integration. The representative standard fingerprint of samples is shown in Figure 1, and the characteristic peaks were labeled based on their elution order (peaks 1–12). Of the common peaks, four were identified by matching retention time (RT) to the respective reference compounds. These were: peak no 4, caffeic acid (14.81 min); no 5, syringic acid (16.90 min); no 6, ferulic acid (23.35 min); and no 10, quercetin (42.62 min). Peak no 7 (RT = 28.40 min) was chosen as the internal reference peak, since it was present as a maximum peak in the middle of the chromatogram. The chosen peak was used for calculation of relative retention time (RRT) and relative peak area (RPA) of the rest peaks which was needed for quality evaluation of fingerprint (data not shown). According to the literature, and based on retention time and chromatographic profile, this is likely to be ferulic acid glucoside (Raal et al., 2012).

### Quantification of phenolic compounds in chamomile samples

ANOVA test showed no statistically significant differences among the chamomile samples based on phenolic compound concentration, nor on color, consistency or manufacturer. The proposed HPLC/UV method was successfully applied to the simultaneous determination of the selected phenolic compounds in chamomile samples. Base on the data summarized in Table 3, the concentration of flavonoids and phenolic acids in chamomile samples represent the following order: quercetin > myricetin > syringic acid > caffeic acid > keampferol > ferulic acid > rutin > gallic acid > p-coumaric acid. Some compounds such as caffeic and syringic acids, and myricetin occurred in a wide range of concentrations, 0.38–3.36 mg/g dry weight, 0.34–3.72 mg/g dry weight and 1.17–2.91 mg/g dry weight, respectively. A different choice of agricultural practice, storage and processing of plant material and final manufacturing procedures will inevitably influence the composition and content of active compounds in the final products (Nováková et al., 2010). The results obtained in this study concur with the findings reported therein which in turn confirms that among the simple phenolics, quercetin, caffeic, and syringic acid have been noted as the main components of chamomile flowers (Mulinacci et al., 2000; Roby et al., 2013). Literature screening shows that only Roby et al. (2013) have identified a number of these, i.e., chlorogenic, gallic, caffeic, p-coumaric and ferulic acids, and quercetin, whereas Nováková et al. (2010) have identified caffeic and chlorogenic acids, rutin, quercetin and kaempferol in methanolic extracts of chamomile. In these papers, quantification was expressed as % and μmol/L, respectively, however, it was decided to recalculate the units and compare the obtained data with the literature. The results obtained for caffeic and ferulic acids by Roby et al. (2013) are for rutin obtained by Nováková et al. (2010) are in agreement with those from this work, while for gallic acid, quercetin, kaempferol, and p-coumaric acid estimated in our chamomile extract was higher than those reported by the previous authors. It could be explained by the differences in growing conditions in different region and also the differences in solvents used for extraction.

**Table 3:** **Results of determination of phenolic compounds and antioxidant activity in chamomile samples (arithmetic mean ± standard deviation)**.

**No**.	**Gallic acid μg/g**	**Caffeic acid mg/g**	**Syringic acid mg/g**	**p-Coumaric acid μg/g**	**Ferulic acid mg/g**	**Rutin μg/g**	**Myricetin mg/g**	**Quercetin mg/g**	**Keampferol mg/g**	**DPPH mmol/L**	**FRAP mmol/L**
1	112.9 ± 1.5	1.14 ± 0.01	1.65 ± 0.01	17.1 ± 1.6	0.06 ± 0.04	173.6 ± 3.8	1.38 ± 0.02	2.37 ± 0.02	0.94 ± 0.01	21.68 ± 0.19	3.08 ± 0.09
2	92.9 ± 0.9	2.87 ± 0.04	3.34 ± 0.03	22.8 ± 1.5	1.45 ± 0.01	195.0 ± 1.5	2.74 ± 0.01	2.33 ± 0.03	0.98 ± 0.06	33.49 ± 1.72	4.82 ± 0.13
3	65.5 ± 1.8	0.57 ± 0.04	0.55 ± 0.01	3.3 ± 3.3	0.20 ± 0.03	151.0 ± 3.2	1.17 ± 0.01	2.08 ± 0.01	0.93 ± 0.01	7.14 ± 0.81	2.44 ± 0.09
4	78.1 ± 1.7	0.39 ± 0.02	0.51 ± 0.02	9.7 ± 1.2	0.22 ± 0.01	226.3 ± 1.8	1.17 ± 0.01	2.04 ± 0.05	0.91 ± 0.03	14.45 ± 0.88	2.08 ± 0.14
5	73.6 ± 2.6	0.61 ± 0.06	0.77 ± 0.02	35.5 ± 3.1	0.30 ± 0.04	315.6 ± 2.0	1.16 ± 0.02	2.12 ± 0.06	1.07 ± 0.02	7.56 ± 1.14	2.00 ± 0.13
6	124.5 ± 0.8	0.38 ± 0.01	0.34 ± 0.04	20.5 ± 0.8	0.09 ± 0.02	271.0 ± 1.7	1.22 ± 0.02	2.02 ± 0.03	1.04 ± 0.03	15.78 ± 2.12	2.77 ± 0.05
7	63.3 ± 5.2	0.39 ± 0.02	0.43 ± 0.03	15.5 ± 0.2	0.16 ± 0.03	293.4 ± 3.2	1.18 ± 0.04	2.08 ± 0.04	0.93 ± 0.05	6.87 ± 1.81	1.56 ± 0.13
8	277.4 ± 2.8	1.15 ± 0.03	0.99 ± 0.06	23.5 ± 3.9	0.38 ± 0.01	272.4 ± 1.6	1.24 ± 0.01	2.16 ± 0.03	0.93 ± 0.02	19.05 ± 3.02	2.63 ± 0.08
9	212.5 ± 1.1	1.34 ± 0.04	0.83 ± 0.03	3.2 ± 0.8	0.31 ± 0.02	151.0 ± 0.9	1.17 ± 0.02	2.08 ± 0.01	0.93 ± 0.01	4.06 ± 1.75	1.84 ± 0.16
10	64.9 ± 2.4	0.85 ± 0.01	1.14 ± 0.02	18.4 ± 1.4	0.39 ± 0.06	194.7 ± 3.7	1.26 ± 0.02	2.12 ± 0.03	0.94 ± 0.01	27.50 ± 1.61	2.70 ± 0.22
11	64.0 ± 2.2	0.83 ± 0.08	1.14 ± 0.01	17.0 ± 1.0	0.40 ± 0.02	215.7 ± 4.8	1.28 ± 0.01	2.14 ± 0.02	0.92 ± 0.04	21.63 ± 1.09	2.63 ± 0.10
12	65.2 ± 3.5	1.56 ± 0.04	1.91 ± 0.03	26.4 ± 1.5	0.60 ± 0.04	239.1 ± 1.3	1.44 ± 0.01	2.32 ± 0.05	0.90 ± 0.01	26.63 ± 1.24	2.67 ± 0.09
13	65.2 ± 8.45	0.57 ± 0.06	0.43 ± 0.01	45.5 ± 0.5	0.19 ± 0.01	185.6 ± 1.4	1.11 ± 0.01	2.02 ± 0.01	0.97 ± 0.02	3.63 ± 1.92	1.80 ± 0.13
14	101.83 ± 0.8	3.36 ± 0.06	3.72 ± 0.01	84.2 ± 1.6	1.48 ± 0.02	269.3 ± 4.6	2.91 ± 0.01	2.53 ± 0.04	0.92 ± 0.02	34.13 ± 4.51	4.33 ± 0.04
15	67.7 ± 2.9	1.54 ± 0.03	2.48 ± 0.02	46.7 ± 1.3	0.82 ± 0.03	205.7 ± 3.6	1.57 ± 0.06	2.39 ± 0.03	1.18 ± 0.02	29.83 ± 0.98	3.55 ± 0.11
16	65.2 ± 3.2	1.23 ± 0.02	2.24 ± 0.02	19.1 ± 1.9	1.31 ± 0.02	206.6 ± 1.9	1.95 ± 0.03	2.72 ± 0.02	0.98 ± 0.03	34.39 ± 1.93	3.85 ± 0.08
17	78.7 ± 1.2	2.33 ± 0.01	2.87 ± 0.05	13.1 ± 2.9	1.27 ± 0.01	199.7 ± 8.7	1.98 ± 0.01	2.57 ± 0.02	1.00 ± 0.03	32.34 ± 0.87	3.23 ± 0.10
18	64.4 ± 0.2	0.77 ± 0.00	0.85 ± 0.06	8.0 ± 2.8	0.28 ± 0.02	331.2 ± 6.1	1.28 ± 0.02	2.13 ± 0.03	0.99 ± 0.01	13.13 ± 1.48	3.04 ± 0.10
19	142.6 ± 2.5	0.53 ± 0.04	0.76 ± 0.04	31.7 ± 1.7	0.29 ± 0.05	323.6 ± 3.7	1.23 ± 0.01	2.10 ± 0.04	1.03 ± 0.06	8.44 ± 2.73	2.12 ± 0.09

### Antioxidant activity

Antioxidant activity was determined using DPPH and FRAP assays and the results are summarized in Table 3. DPPH and FRAP values were found within the range 4.06–34.39 mmol TE/L and 1.56–4.82 mmol Fe^2+^/L, respectively. Generally, samples with high concentrations of phenolic acids and flavonoids, especially caffeic and p-coumaric acids, myricetin, kaempferol and quercetin displayed high antioxidant activity. Similar results in terms of correlation between antioxidant activity and content of simple phenolic acids and flavonols have been reported by Wojdyło et al. (2007), who determined antioxidant activity and phenolic content of 32 common Polish herbs species from the *Lamiaceae* and *Compositae* families.

The results of DPPH could not be compared with data obtained by other authors who have investigated chamomile samples due to the difference in presentation methods, e.g., results expressed as percentage inhibition of DPPH free radical (Atoui et al., 2005; Horžić et al., 2009; Lin et al., 2009) or as milligrams of ascorbic acid per 1 g of dry plant (Buřičovǎ and Rěblovǎ, 2008), while the total reducing power of chamomile measured in FRAP assay was comparable to values obtained by Katalinic et al. (2006) for infusions of chamomile flowers. The value (0.65 mmol Fe^2+^/L) obtained by Pellegrini et al. (2003) for chamomile tea was slightly lower than those obtained in this study. This may be attributed to different solvents (Turkmen et al., 2006; Hayouni et al., 2007; Dutta et al., 2012; Addai et al., 2013; Settharaksa et al., 2014). Berker et al. (2010) express the results of FRAP assays of chamomile as mmol Trolox per g (5.52 × 10^−2^), again making it difficult to compare with the results from this study.

### Chemometric analysis

#### Correlation analysis

The correlation matrix of phenolic compounds and antioxidant activities are shown in Table 4. Strong positive inter-relations (*r* = 0.704–0.936, *p* < 0.05) were established among caffeic, ferulic and syringic acids, myricetin and quercetin. The values of antioxidant potential obtained using DPPH and FRAP assays were correlated positively to each other (*r* = 0.860, *p* < 0.05). The phenolic compounds that displayed significant (*p* < 0.05) correlations with either DPPH or FRAP assays were caffeic, ferulic and syringic acids, myricetin and quercetin. Correlations suggest the crucial role of phenolic compounds as antioxidant constituents in the plant extract. These results are compatible with those found in the literature (Villaño et al., 2007; Wojdyło et al., 2007; Kuś et al., 2014).

**Table 4:** **Correlation matrix (only statistical significant correlations) of determined phenolic compounds and antioxidant activity in chamomile samples**.

	**Caffeic acid**	**Syringic acid**	**Myricetin**	**Ferulic acid**	**Quercetin**	**FRAP**
Syringic acid	0.909					
Myricetin	0.925	0.906				
Ferulic acid	0.897	0.919	0.936			
p-Coumaric acid	0.488	0.498	0.515			
Quercetin	0.704	0.753	0.7303	0.899		
FRAP	0.798	0.813	0.883	0.874	0.742	
DPPH	0.728	0.807	0.823	0.849	0.823	0.860

Li et al. (2009) have found very strong correlation between caffeic and ferulic acids, and DPPH values (*r* = 0.933 and 0.929, respectively) in the root of *Angelicae Sinensis*, while Gamel and Abdel-Aal (2012) have demonstrated significant correlations between caffeic and ferulic acids, and DPPH values (*r* = 0.722 and 0.793, respectively) in barley cultivars. However, as was the case in this study, no significant correlation was found between p-coumaric acid and DPPH values. Antioxidant activity in phenolic compounds depends on the structure and substitution pattern of hydroxyl groups (Sroka and Cisowski, 2003). The relationship between the structure of phenolic compounds, e.g., flavonoids, phenolic acids, tannins and their antioxidant activity has been studied by other authors (Bors et al., 1990; Kim and Lee, 2004; Villaño et al., 2007; Shahidi and Chandrasekara, 2010).

#### Supervised alignment (SA) method

**Figure 2:**
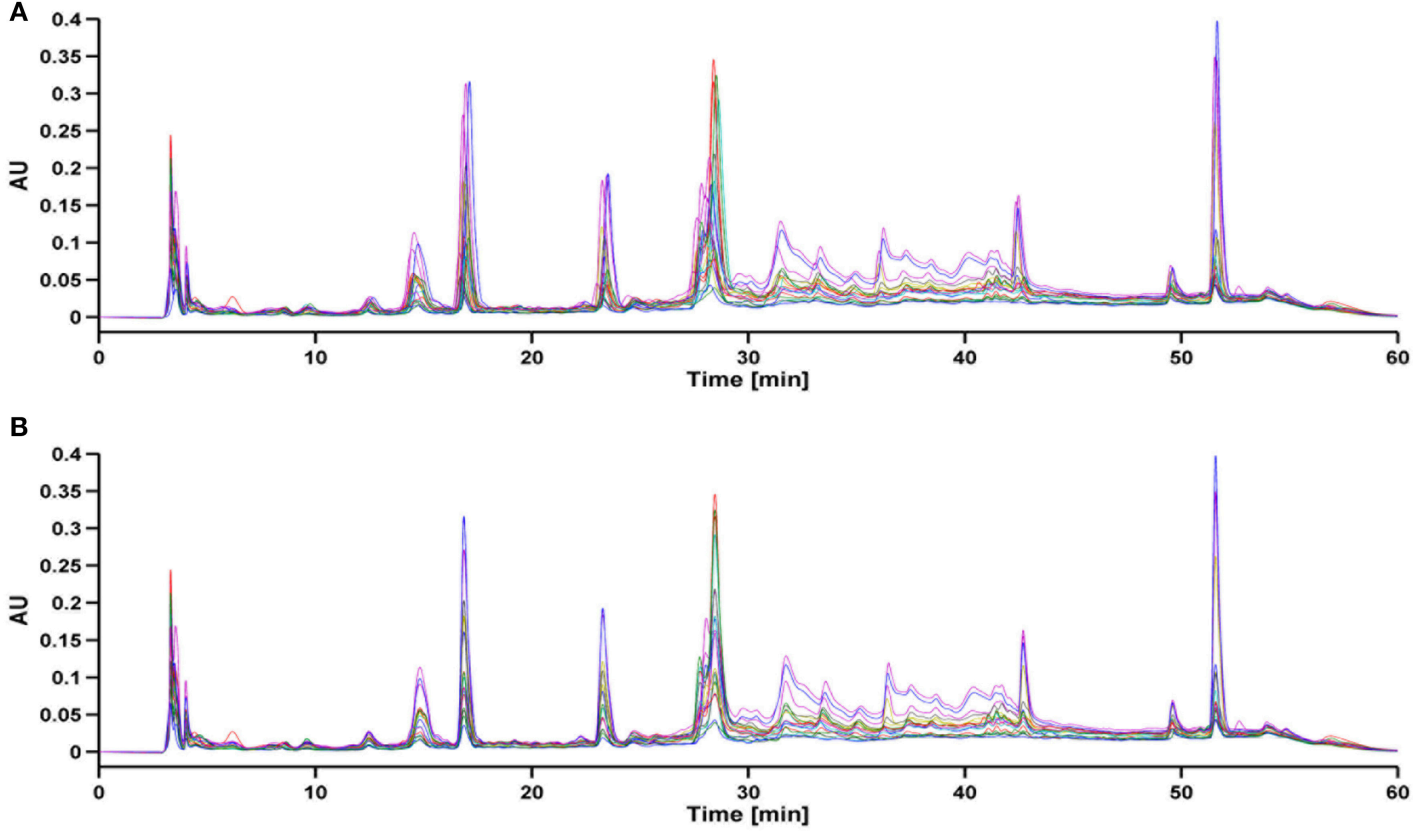
**Chromatograms (A) before warping, (B) after supervised alignment**.

As a result of HPLC/UV analysis, each entire chromatogram was composed of 8250 time points. During the total analysis period, there is a possibility of variations in mobile phase composition, column ageing and instrument instability. Such analytical variability can cause retention time shifts in the peaks detected and impair classification and further determination. Due to the fact that there was a slight retention time shift in the raw data set, those shifts were aligned before any classification techniques were applied. For peak alignment, the Supervised Alignment (SA) method was carried out. Briefly, this method is based on “supervised” selection of common peaks for all chromatograms. The selected peaks are aligned according to the difference in the retention time of selected analytes in each sample and the reference chromatogram. Subsequently, retention times of the fragments between the aforementioned peaks are linearly interpolated. A full description of this method has been explained in detail in the work of Struck et al. (2012). The chromatograms before and after SA are presented in Figure 2. After Supervised Alignment the shifted peaks were satisfactory aligned except for co-eluting peaks (i.e., the region between 28 and 30 min) which were impossible to separate without chromatogram impairment.

#### Similarity analysis

Similarity analysis was performed for entire chromatograms before and after alignment to assess the similarity/dissimilarity of chamomile samples. For this purpose the simulated mean chromatogram was used as a reference and the correlation coefficient was calculated. A correlation coefficient close to 1 suggests a high similarity value between samples. For unaligned chromatograms the regression coefficients varied from 0.834 to 0.976 (data not shown), whereas regression coefficients were better for the aligned data set and ranged from 0.868 to 0.990, as shown in Table 1. These results indicate that the chemical composition in chamomile samples varied slightly even for unaligned chromatograms.

#### Principal component analysis (PCA)

**Figure 3:**
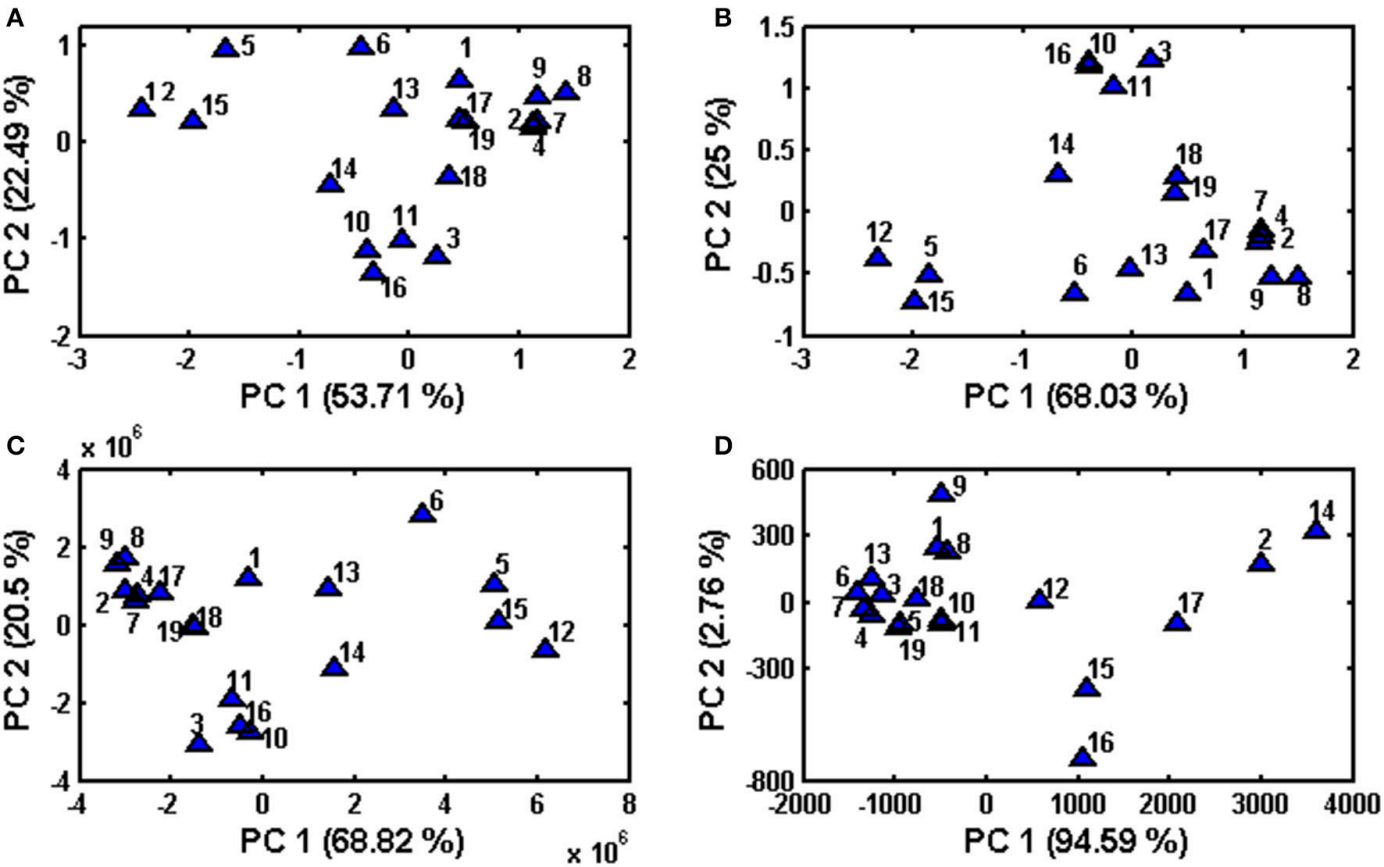
**Principal Component Analysis performed on obtained data sets after column centering (A) raw data set (before alignment), (B) data after supervised alignment, (C) common peaks (peaks 1–12), (D) quantified phenolic compounds**.

PCA is an unsupervised multivariate method that allows reduction of the data matrix, evaluation of samples' clustering tendency and outlier detection based solely on data matrix X (samples vs. variables) (Massart et al., 1997). In PCA no prior knowledge is required of the classification of sample or to which specific group they belong. This method allows data exploration by projection of samples on a hyperplane created by principal components, which is called scores, as well as the contribution of original variables to principal components, which is called loadings. In the present study, score plots were created in order to assess clustering tendency of samples (Figure 3). PCA was performed on entire chromatograms before and after alignment, in addition to the use of areas of common peaks (peaks 1–12) and concentrations of quantified analytes. In regard to Table 1, where chamomile samples are listed according to consistency and color, their projection on principal components scores did not reflect this classification. According to the data itself, the clustering tendency of fingerprints before and after alignment, as well as data containing common peaks (peaks 1–12) is very similar, whereas in the case of data containing quantified analytes the classification of samples is different and samples were grouped according the content of phenolic compounds. Those with the highest levels of phenolics (no 2, 12, 14, 15, 16, and 17) were situated on the right of the graph, those with lower levels on the left.

#### Hierarchical cluster analysis (HCA)

**Figure 4:**
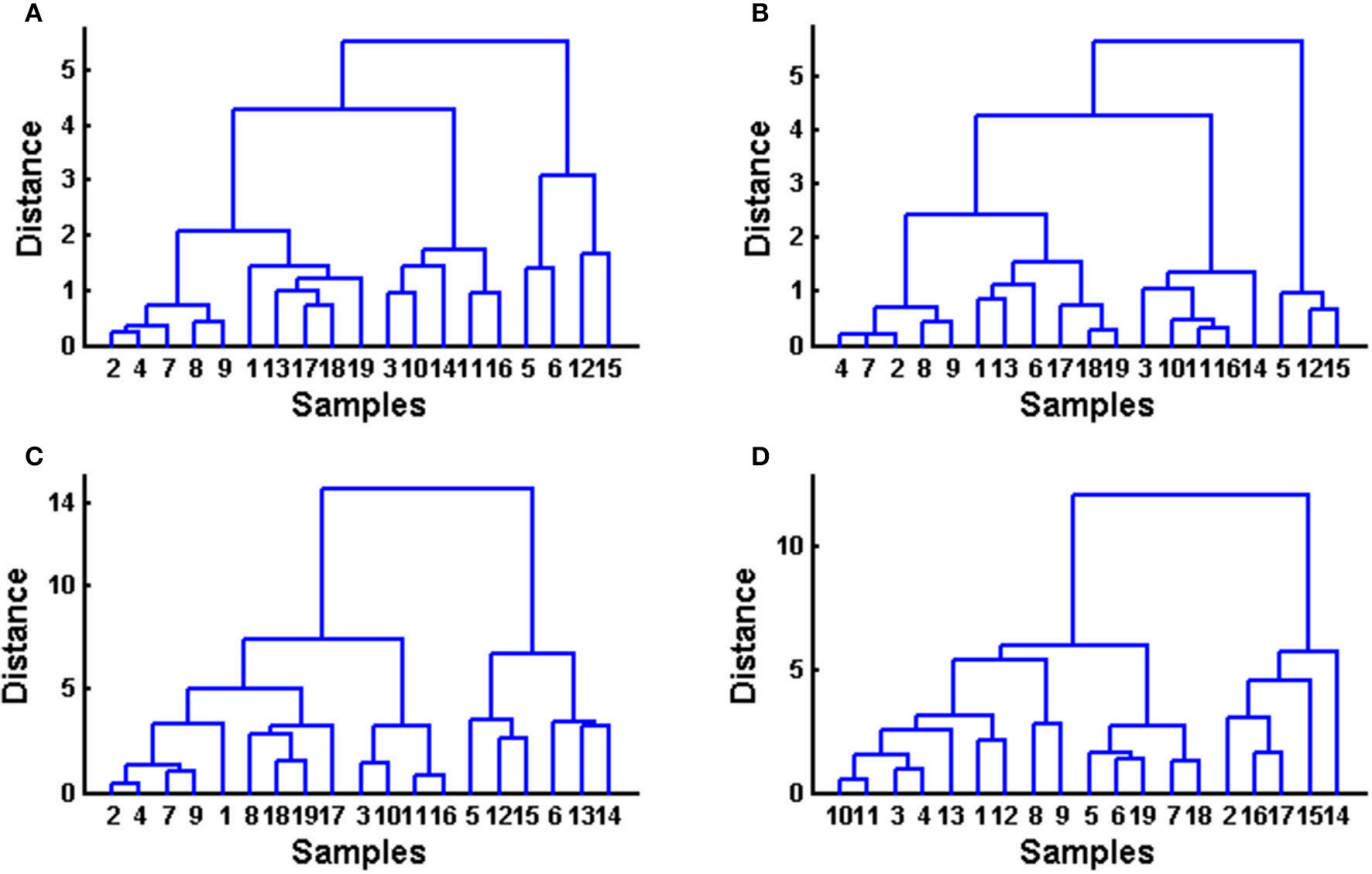
**Hierarchical cluster analysis performed on obtained data sets using Euclidean distance and Ward linkage**. **(A)** raw data set (before alignment), **(B)** data after supervised alignment, **(C)** common peaks (peaks 1–12), **(D)** quantified phenolic compounds.

HCA was performed as a continuation of PCA to assess if, by using a different classification algorithm, it is possible to expect more sensitive sample classification, despite their similarity to each other. As with PCA, HCA is an unsupervised multivariate method, which evaluates the clustering tendency of samples through an iterative process which associates the samples by taking account of the chosen distance between samples and a linkage criterion according to which samples or clusters are merged (Bratchell, 1987). In this study Euclidean distance was checked as a distance similarity measure and Ward's linkage for the entered chromatograms before and after alignment, as well as for common peaks and for quantified analytes among common peaks (Figure 4). Again as with PCA, samples were not separated in HCA into distinct clusters according to color or consistency. Clustering tendency is different according to the data used (entire chromatograms vs. common peaks vs. quantified analytes). Similarly to PCA, the clustering of fingerprints before and after alignment using HCA is very similar. The clustering of a data set containing common peaks is more similar to the clustering of data with entire chromatograms than to data containing four quantified analytes among common peaks but which is different to the aforementioned data sets. In the data set with quantified analytes, some clustering of similar samples according to antioxidative activity value can be observed (e.g., cluster of samples no 2, 14, and 17 and 12, 15, 16 that were measured with the highest DPPH and FRAP values).

## Conclusions

In this study, the chromatographic fingerprinting method combined with quantitative analysis of phenolic compounds was developed based on HPLC/UV. For the fingerprint analysis, twelve “common” peaks were selected to evaluate the similarities of nineteen samples of *Matricaria chamomilla* L. Four of the “common peaks” were identified by comparing their retention times with those of the standard compounds. Statistical analysis indicated that quercetin occurs in chamomile samples at the highest level, with p-coumaric acid occupying the lowest position. Similarities in the chamomile samples were all above 0.868. Correlation analysis showed a strong positive relationship between antioxidant activity and the level of some phenolic compounds. Chemometric analysis indicated that the quality of chamomile samples is not reflected by color or consistency, nor by manufacturer. This study confirms that a combination of chromatographic fingerprint and quantitative analysis can be readily used as a quality consistency control tool for chamomile and related medicinal preparations. The applied strategy seems to be the most promising for the assessment of the investigated plant material.

## Author contributions

AV: planning of investigation, performing the experiments by determination of phenolic compounds, antioxidant activity and HPLC fingerprint. WS: Supervised Alignment method, similarity analysis, principal component analysis, hierarchical cluster analysis. PK: ANOVA test, correlation analysis. MW: scientific consultation, especially in analytical methods. RK: scientific consultation, especially in chemometric methods.

### Conflict of interest statement

The authors declare that the research was conducted in the absence of any commercial or financial relationships that could be construed as a potential conflict of interest.
